# Valedictory Address to the Graduates of the Atlanta Medical College for the Year 1859

**Published:** 1859-11

**Authors:** Eben Hillyer

**Affiliations:** Rome, Ga.


ATLANTA

Medical and Surgical Journal.

Vol. V.] NOVEMBER, 1859. [No. 3.

### ORIGINAL COMMUNICATIONS.

### ARTICLE I.

*Valedictory Address to the Graduates of the Atlanta Medical College for the year* 1859. By Eben Hillyer, M. D., of Rome, Ga.

*Gentlemen of the Graduating Class—*

It is my pleasing duty to appear before you upon this interesting occasion, in behalf of the Faculty of the Atlanta Medical College, to deliver to you the Valedictory Address.

I have called it an interesting occasion, for truly there is much to lend it interest. You have been engaged for several years in preparing for the Medical Profession. Many of you have doubtless had to contend with serious obstacles in the prosecution of your studies. Some of these may, perhaps, have appeared insurmountable; but with stout hearts and determined wills, you toiled on regardless of them; and leaving your homes and your firesides with their loved ones, whose parting words were those of affectionate encouragement, you came among these good citizens, and were welcomed by them to their homes, and to the halls of their cherished institution. Its Faculty received you with a kind greeting. With them for several months you have pursued the paths of Science, endeavoring to cull by the wayside, those flowers whose beauties would adorn and decorate the inner temples of your minds.

You have labored together—they have tried to instruct you in that which was useful—to show you those truths and facts in Science, the knowledge of which makes man to be in God's image.

But your journey together is now ended; you have reached the climacteric of your collegiate course; you have not burned the midnight lamp in vain; you have not contended with poverty in vain. By its chastening influence you have been steeled to continued and persistent efforts for success. But this occasion is made doubly interesting by the assemblage of so many fair ones. Do you not feel more joy, more pride in your triumph, when you have woman's inspiring smile? Is not the pleasure and interest of any occasion increased in the sunshine of her presence?

This, then, is one of the most interesting moments of your lives—there is, perhaps, but one more so—that in which woman will enter with you upon the scene—the occasion when

> "Two souls will be united with but a single thought,
> Two hearts that beat but as one."

It is my intention to speak to you briefly of the profession which you have chosen. It is one of great antiquity; in fact, we may say that there is none more old and honored than it. In the writings of Moses we find mention of the practice of Medicine among the Jews. Heroditus speaks of the art having existed among the priests of Egypt when he visited that country. Assyria has been thought by many learned men to be entitled to the distinction of being the parent of Medicine. With the priests of these countries, the Arts and Sciences, Medicine among the rest, seems to have been deposited.

But we have reason to believe that their practice consisted of but little else than the application of soothing unguents, and other remedies, and that their chief reliance was upon incantations and the dexterous use of the magical arts, such as would impress the people with the idea of their power over the operations of Nature, while the real knowledge they possessed was covered up with mystery and superstition. The science of Medicine is said to have been first introduced into Greece by Chiron, a prince of Thessaly, who lived in the thirteenth century, before the christian era. He was the instructor of Æsculapius, and although he first introduced and practiced his art in Greece, it is to his pupil that, by common consent of antiquity, is ascribed the merit of having first devoted himself to the cultivation of Medicine as a science, and made it a distinct object of pursuit.

His contributions to the art was such as induced his countrymen to pay him divine honors, and to designate him the God of Physic, and to erect temples to him in various parts of Greece. So important a part did he have in the founding of the science, that all practitioners of physic have been called his disciples. To his two sons, Podilirius and Mechaon, descended his skill and his knowledge. So distinguished were they, that they were made no inconsiderable characters in the Iliad by the poet Homer—their names occur side by side with the *sage* Ulysses and the *valiant* Nestor. The principal temples dedicated to Æsculapius were those of Cos, of Cnidus, and of Rhodes. The priests of these temples were practitioners of Medicine, and for a long time were the lineal descendants of Æsculapius, and were called the Asclepiadæ. It was their custom to deposit in the temple a tablet, upon which was inscribed a narrative of every case which was successfully treated by them, including a statement of the symptoms of the disease, and the remedies used in the cure. These temples thus became to a certain extent, schools of Medicine. The science of Medicine and Surgery remained stationary in the hands of the Asclepiadæ for several centuries. But during this period Pythagoras established his school of philosophy at Crotona, and we find honorable mention made of his having advanced the science, by prosecuting the study of Anatomy, and his knowledge of the structure and action of the human frame.

We come now to speak of Hippocrates, who may be said to be the father of *scientific medicine*—for he worked a complete revolution in the art, and it is confidently affirmed, that it is more indebted to his ability and research, for its advancement, than to any single individual. He was said to have been a lineal descendant from Æsculapius in the eighteenth degree. He had access to the records of the Asclepiadæ, and from them, in connection with his knowledge and experience, he gave to the world many works, which, considering their antiquity, may be esteemed as containing much that is true, and principles which are well established. His descriptions of diseases are very correct representations of Nature, and indications of treatment generally rational and practical.

It is not my intention to weary you with the history of Medicine; thus much of its origin, age and antiquity is sufficient to entitle it to your honor and respect. All this while, from the period when the first gleanings of the science could be discovered in the manuscripts of the ancient writers until now, it has withstood all opposition. The great principles of rational medicine have remained pretty much the same, from the days of Hippocrates, Galen and Celsus, and other ancient lights in the profession, down to the present. It is often made an objection to Medicine that it is a system of *speculation*, and hence its conclusions are uncertain ; this objection is founded upon the fact that new theories have been sometimes advanced, which, for a season, have been highly esteemed, but by-and-by have sunk into contempt under the workings of an actual experience. Nay, what is worse, some of these dead theories have again been quickened into life, and made to enjoy an undeserved popularity, till at length exposed, their unworthiness has consigned them to merited oblivion. But these facts do not justify the objection. It should be remembered that *speculation is the parent of theory*, and theory *the parent of science.* But for speculation, the theory of gravitation would never have existed, and but for that theory, the astronomy of Newton would never have been demonstrated. Medicine has proved itself able to shake off the vagaries of mere speculation, while it has retained and established the conclusions of truth.

While it has been subject to the changes and influences of new theories, its great and fundamental doctrines have remained the same. It has withstood the persecutions arising from Ignorance and Superstition, and the direct attacks of new systems of medication. For thirty centuries it has remained the same, and, to-day, among the learned professions, it stands as honorable and distinguished as any we have. None can claim a superiority over it. We may say that it comprehends in its range a wider scope, a more extended field of study than any of the others, for with it is necessarily included several sciences which are separate and distinct. But still, with the physician, they have to be mastered. I do not conceive that I can say too much for the profession as a science, *for Science is Truth.* What would medicine be *without it?* It would be what it is among the empirics, those who practice the healing art as blind groping worms of the dust. Or it would be as with the savage medicine man, whose cures consist of charms and the performance of foolish ceremonies. Next to Divine revelation, Science is entitled to our utmost confidence: *its truths are unerring.*

When the Astronomer, Leverrier, discovered the planet which bears his name, he announced its existence to the scientific world without ever having seen it. His knowledge taught him that in the far distant realms of space, this new world to man existed. He wrote to his friends at one of the universities, pointing out its position in the heavens, its distance from the sun, the frequency of its revolutions, its magnitude, and all the laws by which it was governed.—Obeying his instructions, they turned their telescopes to the constellation indicated, and *behold there was the planet* in the position he had assigned it, and obeying the same astronomical laws! What a *triumph* for *science was here!* It disclosed to this man's intellectual vision the existence of this heavenly body; he was as well convinced of its being in the heavens, as if he had seen it with his natural eyes.

The profession of Medicine, as I said, embraces several separate and distinct sciences, which all blend together as a whole, and go to make up the widely extended and comprehensive field of study, to the complete *mastery of which* the short space of time allotted to man's life, is wholly *inadequate.* How perfectly these separate sciences act in wisdom? How perfectly each one comes into the aid of the intelligent physician in the management of his cases—in his enquiries? Anatomy will instruct him of the abnormal position of any of the organs of the human body, or of their deformity—the exact position of such as are liable to disease; or if it be a surgical case, it tells him of every tissue through which his knife will pass; or, if an injury has been received, Anatomy tells him of its extent, and of its danger. Physiology tells him of the healthy or unhealthy performance of the functions of the machinery deranged—whether he will be able to repair the derangement or the injury, for it may be so extensive as to be beyond the reach of human aid.

But if within man's power to relieve, the Materia Medica furnishes abundant means. Chemistry, with Botany, makes the Pharmacopæa replete with medicinal agents whose every property is well known. The researches in Botany have taught us the medicinal virtues of most of the plants. The science of Chemistry has made known their ultimate elements, extracted their active principles, and taught us their poisonous properties; and from the Mineral Kingdom, Chemistry has filled the Materia Medica with compounds from the simple elements, producing salts of decided medical virtues. You can readily perceive how the Sciences furnish the physician with resources in his investigation, and how important it is for them to be clearly understood by him.—And do you not perceive how impossible it is for the Empiric, who attempts to practice the healing art, to do so successfully? His remedies are those used from hearsay. He knows not their *modus operandi;* he has no knowledge of their applicability. Should the agent he uses be a remedy, he knows not how to modify it to suit the peculiar case; it is all guess work with him. Take, for instance, some variety of Schirrus—for among Empirics we have a host of Cancer curers—he knows not the malignant from the non-malignant disease; he applies his remedies at random; some secret nostrum which he thinks has immaculate virtues; the same remedies he applies to all bad, ugly-looking tumors, without distinction of variety. Nine-tenths of his cases are those of simple ulcer in merely an aggravated form, with no malignant tendency, and which are readily cured by the simplest remedies. He *applies his nostrums;* the ulcer is healed; the case is proclaimed abroad as one of great success; his name is heralded to the world as a great physician —*as one who heals Cancers!!!* His failures are unheard of, and do not detract from his fame. How different with the man of Science! A case is brought him—*a bad, ugly-looking one it is;* he examines it carefully. His first thought is to determine whether or not it is of the class of malignant disease. He takes a particle from the affected part, places it under the microscope; he looks for the cancer cell; the science of microscopy teaches him its exact form and characteristics. If he sees it not, he feels easy; he tells his patient to be of good cheer, his case is one of but little danger, and entirely within the reach of remedies, and that probably he will soon be healed. Perhaps he sees the real cancer cell. He is convinced that his worst fears are realized. His next thought is to determine of what variety of Schirrus, or malignant disease, whether of *simple Carcinoma*, the *Medullary Sarcoma*, whether the *Melanotic variety*, or the *Fungus Hematodes.* The danger of these is fully known to him. He next decides whether the case is sufficiently in its incipiency to be turned over to the Surgeon for extirpation, or that it is useless to harrass the patient with any treatment to effect a cure. His remedies are only those of palliation. He tells his patient to put his house in order, that he has only so long to live. Such is the honest and candid dealing of the regular profession.

I wish now to say a few words of the physician himself. His occupation is an arduous one; he is, in a great degree, cut off from the pleasures of life; his calling is one of sympathy; he moves about among the afflicted and the unfortunate; the whole effort of his life is to do good; acts and deeds of charity are with him an every-day practice; for whose is the hand that ministers to the poor and the destitute in the hours of sickness and affliction? He goes alike to the rich and the poor—to the poor without hope of remuneration. That his fellow-being is sick is sufficient for him to know—he goes to him and makes an honest endeavor to relieve him.

I said, gentlemen, that his life was one of toil. Let us see. Late in the evening he approaches his home; the wife of his bosom greets him upon the threshold—his little ones come around him, and with their childish prattle gladden his heart; his wants have been ministered to by his tender wife, and, finally, he has thrown his weary and languid form upon his couch to sleep. The cares of the day have been forgotten and he soon slumbers. Several hours have elapsed; he turns, and having enjoyed his first sweet sleep, composes himself to the further enjoyment of his rest. *But hark!* the clattering feet of a horse is heard in the distance; he listens—it comes nearer—he begins to fear that it is a messenger, to bid him come. Tired nature causes him to hope—wish that he may pass by. But no, the messenger stops—he calls—the physician answers. The messenger tells him he is called in a case of great emergency, many miles distant; that he has been sent in haste to bid him come quickly. The doctor looks out upon the night; 'tis dark, and bitter cold; he looks again upon his warm and comfortable couch; it evokes him; he dreads to go. But he feels that his duty sternly demands it—he hesitates not—he thinks not of his comfort, but calls for his horse, and flings himself into the saddle and dashes forward to the relief of suffering humanity.

Such is the life of the physician. As an hero, his name is scarcely mentioned. With the world his deeds are not those of the brave. They know him not as *one who fear-*

*lessly faces death*—as one who *never* flees from the dread monster. There are no monuments to tell of his heroism in Trafalgar Square, or in the Place D'Vendome; but still, gentlemen, he is a hero. Not in the commingling of the spears, side by side with Godfrey DeBullion and Richard Couer DeLeon; not at the pass of Thermopylæ, or upon the plain of Marathon, or in the battles of Alva or Balaklava, or upon the fields of Italy, amid the carnage of Magenta and Solferino, but he is found day and night hovering around the couch of the cholera-stricken soldier in the hospitals of Scutari. Upon the fields of battle (after grim war has done its worst) he goes among the mangled and mutilated forms of humanity, upon his mission of mercy, to soothe and to heal the wounded; to bind up the broken limbs of those left in the *path of the world's great heroes.* In the great epidemics of plague and cholera, which have almost depopulated the cities of Europe, he has ever remained at his post—even when the whole atmosphere was laden with death—and bade defiance. Still, he has oftentimes fallen victim, and been a martyr to the duties of his profession.

Several centuries ago, when the plague first appeared in one of the cities of Southern Europe, its malignity was so appalling, death was in every place. It seemed that the city must be depopulated; the physicians were discouraged—in despair, that their efforts to arrest the disease had been unavailing. The remedies which had been used were without effect. They held a council to confer together, and determine upon some plan of treatment which might be successful. All agreed that it was impossible to fix upon any plan of treatment which would promise success, until an autopsy had been made of some of the cases. But who should make it? for all knew that it was certain death to him who undertook the task.

After a slight pause in their deliberations, one of their number arose and announced that he would make the examination. He was in the vigor of his manhood, with all the fair prospects before him—of honor and distinction—and he was fully aware that he was to pay the penalty, for what he was about to do, with his life. That very evening he procured a body which had been a victim to the scourge, and in a retired chamber he made the post-mortem, and noted accurately, upon parchment, all the lesions he observed; and when he had finished, put the manuscript in a vase of vinegar, that the contagion might be made inert, and placed it accesible to his brethren, and calmly awaited death, by which he was overtaken in a few hours. *Could any but an hero do such a deed?* He died, not for his own glory, but for the *salvation of his fellow-beings!* He threw himself into the very jaws of death itself to save his fellow-men.—When the citizens of our own beautiful Savannah were scourged by the Yellow Fever, and were dying by scores, daily, *all that could leave did so.* The Physician and the Minister of the Gospel remained, and gave their utmost energies to the relief of the suffering and dying. *Were not these men heroes?* 'T was disinterested heroism; for they knew that they were not winning honor and glory. Where is the monument reared to tell the fate of Wildman and Gordon? To tell how gloriously they died in the discharge of their duty—to tell posterity how, in the darkest hour of their city's affliction, they did not falter nor desert her, but stood heroically by her, and died faithfully in the discharge of their obligations to her as physicians?

And now, gentlemen, that your course of study is ended, and as you are about to enter the world as Physicians, I wish to speak to you a little of the future. But first let me say, as an entirely disinterested person, that I think you have made a judicious selection of the institution in which to prosecute your studies, and that you will never have cause to regret your selection.

I know that the Faculty have labored zealously to teach you all the truths of the Medical Sciences; and that, in going out into the world with a diploma from them, you are prepared to practice your Art as men of science. Follow the precepts they have set before you, and your professional success is certain. The *greatest efforts of their lives have been to put the Atlanta Medical College upon a high and honorable basis.*

They have spared neither labor or money in its cause.—And to-day, gentlemen, I am *proud to say* that their *energy* and *industry* have not been in vain; for, in the face of the opposition of older and rival institutions, she stands prominent among the institutions of the South, an *ornament* to the city of Atlanta, and the pride of the Physicians of Upper and Middle Georgia.

By authority of the State Legislature, she sends you forth as being fully prepared to practice Medicine and Surgery; and conferring upon you the degree of "Doctor in the Art of Medicine"—giving you a diploma with the signatures of the Faculty, and the seal of the College attached, thus giving evidence that she is responsible for your acts as professional men.

Will you not strive then, gentlemen, to maintain the dignity of your Alma Mater—to reflect credit upon her as well as upon yourselves? Strive to become men of Science; pursue closely the knowledge of your profession. Your studies have not ended, they have just began: cease not to study while you live.

Enter the lists among the practitioners of the healing art, and *fight valiantly for your noble profession*, and let the *great endeavor* of your *lives* be to relieve Scientific Medicine from ignorance, superstition, and the thraldom of empiricism.

